# Feasibility and implementation insights from implementing the health navigator model for cancer prevention among people experiencing homelessness across Europe

**DOI:** 10.1093/eurpub/ckag119

**Published:** 2026-08-13

**Authors:** Katerina Belogianni, Alejandro Gil-Salmerón, Tobias Fragner, Julia Gawronska, Rosa Trenado Gomez, Ioanna Tabaki, Maria Moudatsou, Lee Smith, Tamara Alhambra-Borras, Vicent Blanes Selva, Archontoula Dalma, Matina Kouvari, Igor Grabovac

**Affiliations:** Institute of Preventive Medicine, Environmental and Occupational Health Prolepsis, Athens, Greece; International Foundation for Integrated Care (IFIC), Oxford, United Kingdom; PhD Programme in Social Work, Faculty of Social Work, Complutense University of Madrid, Madrid, Spain; Department of Social and Preventive Medicine, Centre for Public Health, Medical University of Vienna, Vienna, Austria; Centre for Health, Performance and Wellbeing, Anglia Ruskin University, Cambridge, United Kingdom; Foundation for Biosanitary Research and Innovation in Primary Care (FIIBAP), Madrid, Spain; PRAKSIS—Programs of Development, Social Support and Medical Cooperation, Athens, Greece; PRAKSIS—Programs of Development, Social Support and Medical Cooperation, Athens, Greece; Centre for Health, Performance and Wellbeing, Anglia Ruskin University, Cambridge, United Kingdom; Polibienestar Research Institute, University of Valencia, Valencia, Spain; Biomedical Data Science Lab, Instituto Universitario de Tecnologías de la Información y Comunicaciones, Universitat Politècnica de València, Valencia, Spain; Institute of Preventive Medicine, Environmental and Occupational Health Prolepsis, Athens, Greece; Institute of Preventive Medicine, Environmental and Occupational Health Prolepsis, Athens, Greece; Department of Social and Preventive Medicine, Centre for Public Health, Medical University of Vienna, Vienna, Austria; Comprehensive Cancer Centre, Medical University of Vienna and Vienna General Hospital, Vienna, Austria

## Abstract

Cancer-related mortality among People Experiencing Homelessness (PEH) is twice as high as in the housed population, with one-third of deaths in this community linked to conditions preventable through timely healthcare. The Health Navigator Model (HNM) is a person-centred approach to improve healthcare access for PEH. This study assessed the feasibility of implementing the HNM across four European countries. A non-randomised, single-arm pilot study was conducted from June 2022 to December 2023 in Austria, Spain, Greece, and the UK. Trained Health Navigators supported adult PEH—excluding those with cancer diagnoses or cognitive impairments—through tailored guidance, education, and referrals. Feasibility indicators (demographics, recruitment, retention, engagement) were collected at baseline and follow-up points. Health Navigators’ experiences were explored via qualitative interviews. Out of 1981 individuals approached, 652 PEH participated. The average age was 47.4 years; 64.1% were male, 41.6% relied on state benefits and 62.4% had experienced homelessness for over a year. Nineteen Health Navigators (mean age 34.95 years) were involved. Engagement included cancer education (*N = *494), health consultations (*N = *286), specialist referrals (*N = *212), and screening tests (*N = *169). Follow-up rates were 69% at the first time point and 42.5% at the second. Low cancer awareness, prioritisation of subsistence needs over health, and system-level barriers were identified as challenges, while building trust with individuals and stakeholders emerged as facilitators. Health Navigators reported increased empathy, awareness of PEH’s needs, and confidence in empowering them as key benefits of their role. Training skilled Health Navigators, building trust and fostering strong inter-organisational collaborations are key to successfully implementing the HNM among PEH.

## Introduction

Cancer incidence and mortality are rising worldwide. In 2020, over 10 million cancer-related deaths were recorded globally [1], with lung, breast, and colorectal cancers being the most prevalent [2]. Timely cancer diagnosis and appropriate subsequent treatment can increase survival rates for certain types of cancer by preventing the progression of the disease to advanced stages [3]. In many countries, however, timely and equitable access to healthcare is not ensured, particularly for disadvantaged individuals, including People Experiencing Homelessness (PEH) [4]. Homelessness is a multifactorial problem where social (stigmatisation, discrimination, etc.), personal (mental health issues, adverse childhood experiences, personal histories of violence, etc.), and structural (employment opportunities, lack of low-cost housing, lack of health insurance) factors place individuals in a vulnerable situation [5, 6]. In Europe, at least 895,000 people are currently experiencing homelessness [7]. Homelessness per se has been associated with an increased risk of morbidity and mortality from infectious and chronic diseases [6], with evidence suggesting that about one-third of deaths among PEH occur due to causes amenable to timely healthcare [8]. For PEH, immediate survival needs often outweigh preventive priorities. Combined with co-existing mental health issues, drug and alcohol abuse, and exposure to injury or crisis, these circumstances frequently push towards emergency and acute health services, leaving limited time, capacity, and opportunity for health prevention [9].

Cancer-related mortality is twice as high in PEH compared to the housed population [10]. This is mainly due to the aforementioned factors that hinder this population’s access to health services as well as the prevalence of individual cancer risk factors such as unhealthy lifestyle behaviours (tobacco use, alcohol abuse, unprotected sexual behaviour, illicit drug use, etc.) and exposure to environmental risks (air pollution, prolonged sun exposure, etc.) [11]. Cancer screening can prevent cancer-related deaths [12] and has been found to be cost-effective for healthcare systems as a primary prevention intervention and early detection measure [13]. Other cancer prevention strategies include educational and awareness campaigns, as well as the availability and timely access to vaccinations or other medical tests [14]. These strategies can be applied at an individual level and supported by governmental policies, campaigns, and the national healthcare system [14]. Considering the complex needs of PEH and providing them with incentives to access care (e.g. vouchers, transport support), as well as efforts to reduce waiting lists and optimise multidisciplinary healthcare delivery, have been proposed as possible solutions to improve healthcare for PEH and reduce associated healthcare costs [15].

Although clinical guidelines have been developed in the USA and Canada for vulnerable housed populations to improve social and health outcomes [16], European healthcare systems rarely have streamlined cancer support services for this population [17]. In addition, PEH’s subsistence needs for food and shelter, combined with cancer misperceptions and low awareness of cancer symptomatology, may further prevent their participation in cancer prevention and screening opportunities [17]. Patient Empowerment and Patient Navigation are two models proven effective in increasing literacy and patient empowerment in decision-making. The World Health Organization describes Patient Empowerment as the ‘process through which people gain greater control over decisions and actions affecting their health’ [18], while Patient Navigation is a community-based, person-centred model where an intermediate facilitator, the ‘Navigator’, guides and supports individuals to overcome barriers in accessing healthcare services [19].

To the best of our knowledge, there are no European initiatives aimed at ‘bridging the gap’ across the socioeconomic, systemic, and individual determinants that hinder access to healthcare for people facing housing insecurity. In this regard, the European CANCERLESS project—Cancer prevention and early detection among the homeless population in Europe—used the Health Navigator Model (HNM) to address health and social disparities and enhance timely access to and engagement of PEH in primary and secondary cancer prevention services [20]. The Health Navigator Model is a person-centred integrated care model [20], embedding elements from the Patient Empowerment Model and the Patient Navigation Model and implemented as a real-life intervention among PEH.

The project was implemented in four European countries—Greece, Spain, Austria, and the United Kingdom (UK)—to reflect diverse social, policy, and service contexts in which homelessness is experienced and addressed. These countries were selected to capture variation in homelessness patterns, migration-related pressures and the organisations of health and social care services, thereby allowing the programme to be explored across different implementation settings. In Greece, homelessness has been shaped by the combined effects of economic instability and refugee reception pressures [21]. In Spain, rising housing costs, housing shortages, and migration flows have contributed to increasing homelessness [22]. In Austria, homelessness persists, with approximately 20,000 registered PEH [23]. Finally, in the UK, homelessness remains a major social issue, with 4793 people recorded as sleeping rough in 2025 in the context of sustained housing insecurity and cost-of-living pressures [24].

Real-life interventions are essential for assessing the feasibility and efficacy of community-level outcomes and for preventing waste of resources and time before scaling up [25]. Testing the feasibility of a real-life intervention can provide insights into recruitment capacity, data collection procedures, the acceptability and suitability of the intervention, including participants’ engagement with its components, and any issues that hinder its implementation [26]. These considerations are particularly important for populations facing health disparities and complex needs, as they help identify necessary adaptations for this specific population [27].

To this end, this study aims to report feasibility indicators of the Health Navigator Model as part of the CANCERLESS project and to inform policymakers and researchers in designing and implementing similar large-scale initiatives by addressing the following research questions: (i) How many PEH were approached and participated in the project? (ii) How many PEH completed the intervention and complied with the data collection protocols? (iii) What navigational services were offered to PEH, and to what extent did PEH engage with the interventional activities? (iv) What were the main barriers and facilitators encountered by Health Navigators during the implementation of the intervention, and how were these addressed?

## Methods

### Design and target population

A non-randomised, single-arm pilot implementation study (trial NCT05406687) was conducted to evaluate the feasibility of applying the HNM among PEH, as defined by the ETHOS typology [28]. Due to ethical concerns and difficulties accessing this population, no control group was included. Eligible participants were adults (>18 years) from any ETHOS category, excluding those with cognitive disabilities, confirmed cancer, inability to consent, or barriers such as severe mental health crises, intoxication or major language issues. The target sample size was 1500 PEH across Austria (200), Spain (400), Greece (400), and the UK (500). Because this was a pilot study guided by implementation science frameworks, no formal a priori sample size calculation for effectiveness outcomes was conducted. Instead, recruitment targets were determined pragmatically in consultation with participating countries, reflecting the operational realities of delivering the intervention and enabling evaluation of feasibility and implementation processes across settings. Ethical approval was obtained from the Medical University of Vienna and the local ethics boards; participation was voluntary, with informed written consent. Given the absence of established benchmarks for this population, feasibility was interpreted in terms of implementation viability, reach, and identification of barriers and facilitators rather than predefined success thresholds.

### Intervention

#### Health navigators

Health Navigators (HNs) were recruited through health and social care organisations providing services to PEH. Eligible candidates included professionals with health or social care backgrounds, as well as individuals with previous experience of homelessness (peer Navigators) who were already working in or were affiliated with each pilot site [29]. Prior to providing any services, all HNs underwent structured training to enhance their understanding of cancer-related issues, communication skills, and knowledge of local health and social service systems, including how to establish trust with service users and stakeholders [29]. Navigators were trained to offer broad and person-centred services, ensuring that participants would receive comprehensive, practical and emotional assistance throughout their care journey.

#### Pilot sites

The HNM was implemented regionally in Austria (Vienna), Spain (Madrid), Greece (Attica, Thessaloniki) and the United Kingdom (UK) (Cambridge, King’s Lynn). Social and day-care centres (Spain, Greece, UK), outpatient clinics (Vienna), and shelters for PEH (UK) were used as pilot sites to reach potential participants and/or to provide navigational services, as appropriate.

#### Data collection and monitoring

The pilot implementation study started in June 2022 and ended in December 2023, with an overall study duration of 19 months. There were three data collection time points: (i) before entering the study (baseline), (ii) 4–6 weeks after entering the study (first follow-up) and (iii) when exiting the study (second follow-up). Throughout the 19-month intervention period, each pilot site designated a Coordinator responsible for monthly reporting. These included completing a structured Google Form that documented the numbers of participants approached, recruited, and retained, as well as the types of activities undertaken by participants. To support ongoing implementation, monthly virtual meetings were held with Coordinators and HNs to review challenges, share operational updates, and plan next steps. At the study’s conclusion, Coordinators submitted a final report detailing overall feasibility metrics and a summary of the navigational services delivered at each respective site. Additionally, Health Navigators (*n* = 11) were invited to participate in semi-structured interviews. Interviews were conducted at each site either face-to-face or online by trained research staff. A common interview guide was used across sites and was forward- and back-translated into the local languages. Interviews lasted between 15 min and over 1 h.

### Feasibility outcomes

This paper reports on the feasibility outcomes of the intervention, not its effectiveness or acceptance by PEH. Key feasibility measures included recruitment capacity (number of PEH approached and consented), data collection and retention rates (questionnaire completion and reasons for dropout), and baseline demographic and socio-economic data of PEH and Health Navigators (HNs). The study also documented navigational services provided by HNs and cancer prevention activities attended by participants. Health Navigators’ experiences in their roles, the challenges encountered during implementation, and the adaptations made to address them were also explored.

### Data analysis

Descriptive statistics were computed, including figures and tables, to present the number of participants (*N*) and/or proportion of participants (% of total *N*) for each feasibility outcome. Qualitative interviews were audio-recorded, transcribed verbatim and translated into English where needed. An initial coding framework was developed centrally and shared with each site, then refined to reflect context-specific data. Transcripts were coded using MAXQDA software. Data were analysed using thematic analysis, combining deductive coding based on pre-identified feasibility domains with inductive coding to capture emergent issues. Cross-country findings were merged iteratively through discussions among researchers from different local contexts, refining the analysis to create a comprehensive thematic framework.

## Results

The study enrolment flow diagram for all participants is presented in Supplementary Fig. S1. In total, more than 1981 PEH were approached and informed about the CANCERLESS project. Of those, 1208 (61.0%) did not respond to the invitation, and 121 did not proceed with the project, either because they were ineligible (as decided by the HNs) or for other reasons (e.g., death). As a result, 652 participated in the study. Of those, 450 (69.0%) completed the first follow-up, and 277 (42.5%) completed the second follow-up assessment (Supplementary Fig. S1).

Recruitment patterns varied by country and season across the pilot sites. In total, 142 participants were enrolled in Austria (final recruitment in September 2023), maintaining a consistent monthly rate of 7–11 participants. Spain recruited 197 participants (final recruitment in August 2023), with the majority enrolled during the first 6 months (1–22 per month). In Greece, 158 participants were recruited (final recruitment in November 2023), predominantly in the last 6 months (0–30 per month), while in the UK, 155 participants were recruited (final recruitment in October 2023), with most enrolled in the final 9 months (2–16 per month). Data retention was a significant challenge: 375 of the 652 participants (57.5%) did not complete follow-up assessments or were lost to follow-up. Of these, 64.5% dropped out due to loss of contact or relocation (*n* = 112), loss of interest (*n* = 100), hospitalisation or death (*n* = 13), disengagement from pilot site services (*n* = 12), a new cancer diagnosis (*n* = 2), or other reasons (*n* = 3). The remaining 133 participants (35.5%) continued receiving navigational support despite incomplete follow-up data. Table 1 presents the number of participants (*N*) who completed data at each time point of the study, per country pilot site(s), including those who were lost to follow-up.

**Table 1.** Number of participants (*N*) who completed data at each time point of the study per country pilot site(s), including those who were lost to follow-up

Pilot sites	Participants (*N*) who completed data at each time point (% retention rates)			Participants (*N*) who did not complete data or were lost to follow-up (%)
Timepoints	T0	T1	T2	
Austria	142	81 (57.0)	66 (46.5)	76 (53.5)^a^
Spain	197	114 (57.9)	35 (17.8)	162 (82.2)^b^
Greece	158	145 (91.8)	111 (70.3)	47 (29.7)^c^
UK	155	110 (71.0)	65 (41.9)	90 (58.1)^d^
Total	652	450 (69.0)	277 (42.5)	375 (57.5)

a *n = *65 lost contact/relocated; *n* = 7 loss interest; *n* = 1 diagnosed with cancer; *n* = 1 got health insurance; *n* = 2 no interpreter available.

b *n* = 69 received navigational services without completing data at follow-ups; *n* = 65 loss interest; *n* = 25 lost contact/relocated; *n* = 3 hospitalized or dead.

c *n* = 18 lost contact/relocated; *n* = 11 loss interest; *n* = 9 received navigational services without completing data at follow-ups; *n* = 8 hospitalized or dead; *n* = 1 stopped using services.

d *n* = 55 received navigational services without completing data at follow-ups; *n* = 17 loss interest; *n* = 11 stopped using services; *n* = 4 lost contact/relocated; *n* = 2 hospitalized/dead; *n* = 1 was diagnosed with cancer.

Table 2 presents the demographic and socio-economic characteristics of both Health Navigators (HNs) and people experiencing homelessness (PEH) involved in the study. A total of 19 HNs were engaged across Austria (*n* = 4), Spain (*n* = 3), Greece (*n* = 8), and the UK (*n* = 4), with an average age of 34.95 years. Most held at least a bachelor’s degree (42.1%) or a postgraduate degree (47.4%), and the majority had professional backgrounds in the social sciences, with an average of 6 years of experience. Over half had prior training or experience working with PEH, although participation fluctuated due to staff turnover. Among PEH participants, the mean age was 47.4 years, with 64.1% identifying as male. While 58% had completed secondary education, 34.7% reported no income, and 41.6% depended on state benefits. Although most were national citizens, roughly one-third were refugees, asylum seekers, or lacked legal documentation. Just over half (54.8%) had active health insurance, and 62.4% had experienced homelessness for more than 1 year. Regarding the housing situation, 26.1% were staying in night shelters, 22.1% resided in insecure settings, and 14.1% were rough sleeping.

**Table 2.** Demographic and socio-economic characteristics of total PEH and HNs who participated in the pilot study

Variable	PEH (*N = *652)	Health navigators (*N = *19)
Age (years), mean (±SD)	47.4 (13.8)	34.95 (8.79)
Gender, *N* (%)		
Male	418 (64.1)	5 (26.3)
Female	230 (35.3)	14 (73.7)
Non-binary/other	3 (0.5)	
Prefer not to say	1 (0.2)	
Education level, *N* (%)		
Early childhood or primary education	121 (18.6)	
Low or upper secondary education	378 (58.0)	2 (10.5)
Post-secondary non-tertiary or short-cycle tertiary education	64 (9.8)	
Bachelor or equivalent education	56 (8.6)	8 (42.1)
Master or higher education	5 (0.8)	9 (47.4)
Income source, *N* (%)		
None	226 (34.7)	
Income from work	72 (11.0)	
State benefits	271 (41.6)	
Other source of income	83 (12.7)	
Legal status, *N* (%)		
National citizen	407 (62.4)	
Not having legal documents	119 (18.3)	
Asylum seeker	99 (15.2)	
Refugee	12 (1.8)	
Time experiencing homelessness, *N* (%)		
Up to 3 months	86 (13.2)	
3 months to 1 year	144 (22.1)	
1 to 3 years	120 (18.4)	
3 to 5 years	85 (13.0)	
More than 5 years	202 (31.0)	
Housing situation (ETHOS category),^a^ *N* (%) (*n* = 5 missing)		
Living rough	92 (14.1)	
Staying in a night shelter	170 (26.1)	
Staying in accommodation for the homeless	138 (21.2)	
Staying in women’s shelter	34 (5.2)	
Staying in accommodation for immigrants	3 (0.5)	
Due to be released from institutions	2 (0.3)	
Receiving long-term accommodation support	19 (2.9)	
Living in insecure accommodation	144 (22.1)	
Living under threat of eviction	1 (0.2)	
Living in temporary/non-conventional structures	24 (3.7)	
Living in unfit housing	15 (2.3)	
Living in extreme overcrowding	5 (0.8)	
Health insurance, *N* (%)		
Yes/active^b^	357 (54.8)	
Navigators’ profession, *N* (%)		
Social worker		9 (47.4)
Psychologist		3 (15.8)
Medical doctor or student/nurse		4 (21.1)
Health researcher/other		3 (15.8)
Years of professional experience, mean (±SD)		5.5 (5.6)
Years of experience working with PEH, mean (±SD)		3.6 (4.1)
Had previous training focused on PEH, *N* (%)		12 (63.2)

a Groups according to ETHOS European Typology of Homelessness and housing exclusion categories, available at: https://www.feantsa.org/download/ethos2484215748748239888.pdf.

b In Madrid, 80.7% of PEH had national health insurance (as national citizens or insurance for foreigners). In the UK, all PEH (100%) had health insurance, as nationals, migrants and asylum seekers can register with a local General Practitioner surgery and access the National Health System free of charge.

Health Navigators provided a range of navigational services, including helping participants complete administrative forms (e.g. financial or service access forms), arranging interpretation services, arranging and coordinating medical appointments, accompanying participants to appointments, facilitating communication with health and social care providers and supporting participants’ immediate needs such as access to food or shelter, often in liaison with community organisations (e.g. social kitchens). A detailed list of navigational services provided in each country is presented in Supplementary Table S1.

**Figure 1.**
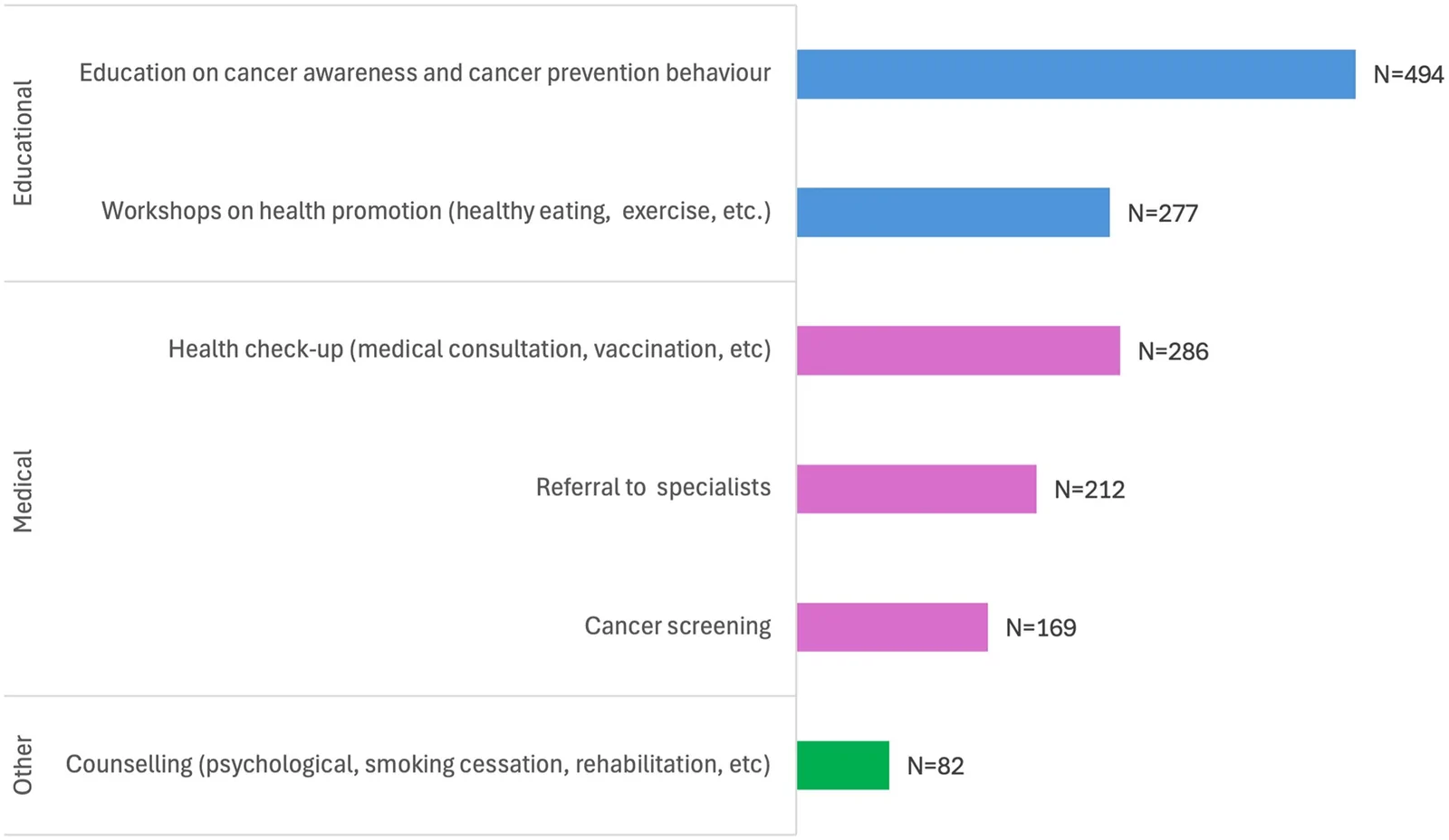
Cancer prevention activities attended by PEH (*N*). Number of people experiencing homelessness who attended cancer prevention activities during the intervention, grouped by educational, medical and other activities.

Cancer prevention efforts encompassed educational, counselling, and medical services, reaching a total of 494 PEH who received information on cancer risks and preventive behaviours—such as sun safety, vaccination and sexual health. In addition, 277 individuals participated in health promotion workshops, while 286 underwent general health assessments, including medical consultations and vaccinations. Referrals to specialists were made for 212 participants, further supporting continuity of care. A total of 169 cancer screening tests were conducted for colorectal (*n* = 59), cervical (*n* = 50), breast (*n* = 23), Hepatitis C (*n* = 18), skin (*n* = 11), prostate (*n* = 6), lung (*n* = 1), and pineal cancer (*n* = 1). Finally, 82 PEH received personalised counselling, addressing mental health (*n* = 31), hygiene (*n* = 21), smoking cessation (*n* = 21), and other concerns such as weight management or rehabilitation (*n* = 9). The number of PEH who engaged in cancer prevention activities is shown in Fig. 1.

The main systemic and individual barriers encountered by Health Navigators during the implementation of the pilot study, as well as the actions and adaptations undertaken to overcome those, are summarised in Table 3. In addition, the following two themes emerged from Health Navigators’ experiences:

**Table 3.** Barriers reported by Health Navigators and actions undertaken to facilitate the implementation of the pilot study

Domain	Barriers encountered during the implementation of the pilot study	Actions/adaptations undertaken during the implementation of the pilot study
Systemic	Disjointed social and healthcare systems.	Health Navigators coordinated tasks and appointments and facilitated information-sharing between social and healthcare units.
	Limited access to healthcare services (e.g. due to lack of health insurance, lack of contact details).	Health Navigators liaised with NGOs offering healthcare services and with private health insurance companies for support.
	Medical staff/systems not prioritising cancer prevention over treatment.	Health Navigators met with medical staff to increase awareness of the importance of cancer prevention and screening for PEH.
Individual	Cancer not a priority compared to subsistence needs (food, shelter).	Health Navigators tried to address participants’ subsistence needs as a first step (whenever possible).
People Experiencing Homelessness	PEH had neither the time nor the willingness to participate.	Health Navigators increased awareness of cancer prevention via education or counselling to enhance participation.
(PEH)	PEH were embarrassed to answer personal questions (e.g. on sexual health, drug use, mental health).	Health Navigators used quiet, private rooms; they built rapport and asked questions with a non-judgmental attitude.
	PEH had a fear of a potential cancer diagnosis.	Health Navigators used alternative terms (e.g. health promotion) instead of “cancer” and provided a clear treatment plan in the event of a cancer diagnosis.
	PEH had low trust in social and healthcare systems.	Health Navigators built trusting relationships with PEH and followed a person-centred, trauma-sensitive approach.
	PEH skipped or cancelled their medical appointments.	Health Navigators made efforts to increase appointment attendance (frequent call reminders; accompaniment to appointments; reimbursement for commuting to attend appointments).
Health Navigators	Difficulties in approaching PEH due to language barriers, PEH’s mental health issues, etc.	Training of Health Navigators to build capacity, use of interpreters and support by skilled (peer) staff to approach PEH.
Other (e.g. operational)	Closure of sites during bank holidays.	No actions.
	Distance to approach sites.	
	Shortage of staff and Health Navigators (e.g. sick, annual leave).	
	Weather conditions limiting access to pilot sites.

1. Low awareness, other priorities and system fragmentation were prominent implementation barriers
  Health Navigators identified several barriers faced by participants, including low cancer awareness, fear of a possible cancer diagnosis and the prioritisation of subsistence needs over health. They also emphasised the need to advocate for cancer prevention among healthcare professionals, who often prioritised secondary care over primary prevention. Additionally, fragmented coordination between social and healthcare services was highlighted as a barrier, limiting appointment coordination, information sharing and access to healthcare.
  *‘Τhere is not enough time available from people. They need other more immediate needs to be met, such as food and sanitation services. Most of the participants also did not want to hear about the cancer. They found it difficult to talk about this disease’ (HN, Greece).*
  *‘Users are very friendly and respectful and willing to make a change, but a big issue was the noncompliance with an agreed appointment, which made the whole process more difficult, leading to losing the bonding with the consequence of higher dropout rates’ (HN, Austria).*
  *‘Main barrier to access are the administrative staff of the health centres and the resistance of some homeless people due to social exclusion variables’ (HN, Spain).*
  *‘I approached multiple organisations, but due to being understaffed they do not want to get involved because they feel like they won’t be able to fulfil their role within the project’ (HN, UK).*
2. The impact of the health navigator role with building trust being the key implementation facilitator
  Health Navigators reported that their role involved multiple tasks and responsibilities. They highlighted that building trusting relationships with participants and partner organisations was an essential component of successful implementation. They reported feeling respected in their roles, and noted that fostering trust with participants contributed to more respectful interactions with healthcare services and increased uptake of the support and services offered. Beyond the positive impact on participants, Health Navigators reflected on the personal benefits of their role, including enhanced cancer awareness, greater empathy towards participants’ needs, improved communication skills and confidence in empowering participants to engage in cancer prevention.
  *‘I believe that during the intervention there was good communication because of the trust that the beneficiaries have in the organisation and the social service where I work. At the same time, the relationship of trust that is established helps in better communication’ (HN, Greece).*
  *‘As a volunteer doing the workshop, there are definitely a number of things we can learn from this. It taught us the bigger picture, challenges they (i.e. PEH) have that may be a barrier in learning, how to approach them, using the right language’ (HN, UK).*
  *‘Homeless people were showing their interest and sharing their knowledge about the CANCERLESS project with other homeless people’ (HN, Spain).*
  *‘I think that the patients benefited a lot from it [the intervention], and because they were able to talk about their problems in peace, there was also enough time for counselling. I think on the whole it was very well received’ (HN, Austria).*

## Discussion

The CANCERLESS pilot study pragmatically applied the HNM to improve PEH access to cancer prevention services. It provided data on the social and health determinants of PEH across Europe, as well as key feasibility outcomes, to guide similar future interventions.

Regarding the social characteristics of the target population, the pooled outcomes indicated that most participants were predominantly male, middle-aged, living on benefits or unemployed, and educated up to a secondary level. Recent data from the USA showed similar trends among 5717 people who experienced homelessness, with most being male (61%), unemployed (50%), and having completed high school (29%) [30]. Participants were most commonly housed in shelters, accommodation for PEH, or living in insecure housing; however, this may be affected by the selected recruitment sites and local factors such as social services, family ties, and climate. Over half had experienced homelessness previously, either short- or long-term. Most were citizens of their respective countries; however, about half lacked health insurance. Ensuring health insurance coverage before implementing cancer prevention is crucial to enable access to healthcare, screening, and treatment for vulnerable populations [31].

Recruiting PEH was challenging, with about half of those invited responding and one-third participating. Barriers included urgent survival needs, low prioritisation of cancer and fear of diagnosis. Future projects should frame cancer prevention within a broader health promotion and awareness context to improve engagement. A low level of knowledge about cancer and its risk factors among PEH has been reported elsewhere [32], indicating the need to increase cancer awareness in this population. Nearly all PEH received tailored or group education to raise cancer awareness, which motivated many to schedule specialist appointments and undergo further screening. Over 160 cancer screening tests, mainly for colorectal and breast/cervical cancers, were completed, demonstrating the intervention’s relevance for this high-risk population.

Despite HNs’ efforts to improve health literacy and reduce barriers—such as providing appointment accompaniment and addressing housing needs—appointment adherence remained low, with many participants dropping out. Similar studies report that about 75% of PEH miss at least one cancer treatment appointment [33]. Service design, delivery, and staff skills affect attendance and medication adherence in this group [34]. Other studies have shown that oncologists often do not adapt consultations (e.g. longer sessions, trauma-informed care) or communication to meet the needs of PEH [35, 36]. This study provided training only to HNs; future interventions may benefit from including medical and care staff in such training. The findings reinforce that individual-level navigation cannot substitute for structural solutions but may serve as a complementary mechanism within broader system-level responses.

The HNM demonstrated partial feasibility in reaching and engaging a subpopulation of PEH under real-world conditions, but was constrained by substantial structural, organisational, and participant-level barriers. Successful implementation appears contingent on strong inter-organisational collaboration, sustained trust-building, and broader structural support. Countries with coordinated staff and stakeholders saw faster implementation through coordinated appointments and information sharing, emphasising the importance of intersectoral collaboration between social and healthcare services. Since primary care often addresses cancer only when symptoms appear, advocating study goals to healthcare providers was crucial to improve cancer prevention access. Similar qualitative findings show that PEH prioritise survival needs over health; thus, consideration of these behavioural determinants by health professionals is important for increasing adherence to health interventions [37]. In this study, HNs’ ability to identify the social determinants of PEH and build trust was critical to success.

Study limitations included the exclusion of participants with severe psychiatric disorders, which limits the generalizability of the findings and may have reduced the representation of a subgroup with particularly high and complex needs and the principle of proportionate universalism. The heterogeneity across recruitment sites is another limitation of the study. Because the programme was implemented in diverse settings (social centres, shelters, clinical sites), feasibility may have varied according to local organisational context, service structures, and participant needs. This should be considered when interpreting differences in recruitment and follow-up across sites. In addition, feasibility was not assessed against pre-defined quantitative thresholds, such as target participation or retention rates. Instead, it was evaluated descriptively through implementation indicators, including service uptake, engagement, follow-up completion and barriers encountered during implementation. Finally, the high number of participants unwilling to disclose or provide information at data-collection points may have led to an underestimation of completion rates, as some participants continued to use navigational services without completing the study questionnaires.

Despite lacking the methodological rigour of a randomised controlled trial, the implementation of the HNM to prevent cancer among PEH in real-life settings was a dynamic process in which actions and adaptations were taken to resolve the barriers encountered throughout the implementation period, following a person-centred and trauma-sensitive approach. As a result, many PEH benefited from the model, setting the stage for a long-term trajectory of similar HNMs focusing on cancer prevention.

## Supplementary Material

ckag119_Supplementary_Data

## Data Availability

The individual-level data underlying this article cannot be shared publicly or with external parties because they contain sensitive health-related information and are subject to ethical, legal, and data protection restrictions. Aggregated, non-disclosive outputs and analysis code may be made available from the corresponding author upon reasonable request, where consistent with the study approvals and applicable data protection requirements. Key points The HNM is a feasible, person-centred approach to improving cancer prevention and care access among PEH. Trained HNs built trust and provided tailored education, referrals, and support, engaging over 650 PEH across four European countries. Effective implementation requires a phased approach including preparatory relationship-building, trauma-sensitive communication, and clear care pathways. Intersectoral collaboration and mapping of local health and social services are essential to overcome systemic barriers to cancer care for PEH. Public health policy should integrate navigation models like HNM to reduce health disparities and promote equity in cancer prevention for vulnerable populations.
