# Supplementary material for: Feasibility and implementation insights from implementing the health navigator model for cancer prevention among people experiencing homelessness across Europe
Source: Eur J Public Health. 2026 Aug 13;36(5):ckag119. doi: 10.1093/eurpub/ckag119 (PMC13470432; doi:10.1093/eurpub/ckag119)
Supplement: Supplementary file 1 [file ckag119_supplementary_data.zip › ejph-2025-06-om-0458-File003.docx]

Supplementary Table 1. Tasks performed by Health Navigators in each country to support users (i.e. people experiencing homelessness) during the implementation of the pilot study

***Austria***

- Interpreting services (Bosnian, Bulgarian, Chinese, Croatian, English, Romanian, Russian, Serbian, Slovak) for example, during consultations with social workers.
- Helping users filling out official forms.
- Accompanying users to the health insurance company and supporting them with Romanian interpreting.
- Arranging medical appointments for users.
- Reminding users of their appointments (internal or external) via accorded modes of contact.
- Organising and facilitating events for health and social care staff at AmberMed.
- Holding multiple one-on-one informational meetings with healthcare providers and staff detailing and developing on-the-ground CANCERLESS activities.
- Providing users with information on (cancer) preventive healthcare and specific recommendations on preventive examinations in both verbal and written form.
- Arranging the availability of resources (e.g. cookbooks, public transport tickets) to support users in understanding recommendations.
- Liaising with external supporting services or preventive healthcare services (in person or via telephone).
- Liaising with internal (AmberMed) supporting services and additional programmes (e.g. “social prescribing”).
- Liaising with internal (AmberMed) healthcare providers on users’ needs and participation in CANCERLESS activities.
- Organising multiple “open door” events with general health consultations at the facilities of Caritas Austria.

***Spain***

- Providing users with information on (cancer) preventive healthcare and specific recommendations on preventive examinations.
- Holding one-to-one meeting to assess medical needs and develop a plan of CANCERLESS activities.
- Providing health education and motivational interviewing to empower users towards screening for preventable cancers.
- Supporting users to obtain health coverage and access to other health and social benefits.
- Arranging and managing users’ hospital appointments.
- Accompanying users to medical appointments (i.e. for cancer screening).

***Greece***

- Providing users with information on (cancer) preventive healthcare and specific recommendations (e.g., protection from sun exposure, dangerous sexual behaviour, use of substances, smoking cessation, diet, hygiene and physical activity).
- Providing users with sanitation and other essentials (medicines, clothes, etc.).
- Psychosocial support (e.g., about family relationships).
- Counselling and support on financial management.
- Support users to access social services such as night shelters, soup kitchens, social hairdressers and other society sectors for accommodation.
- Supporting users to fill out official forms (e.g., for tax requests, allowance, identity card).
- Supporting users with obtaining apartment equipment.
- Job counselling and referral to organisations for further support.
- Providing users with legal advice from organization’s lawyer and referral to other civil society sectors.

***United Kingdom***

- Educating users about cancer, cancer prevention and detection of cancer symptoms.
- Supporting users registering with local General Practitioner services.
- Arranging and reminding users of their medical appointments.
- Providing reimbursement (for taxis or bus fares) to attend medical appointments.
- Weekly cooking at Purfleet Trust during the first six months of the intervention.
- Supporting participating organisations to receive leaflets on different types of cancer, free condoms and sexually transmitted infection screenings tests.
- Organinising and supporting users to attend physical activity classes available at Purfleet (swimming, wellbeing walks, football and gym).
